# Editorial: Therapeutic potential of the cannabinoid CB2 receptor

**DOI:** 10.3389/fphar.2022.1039564

**Published:** 2022-10-07

**Authors:** Reem Smoum, Uwe Grether, Meliha Karsak, Andrea J. Vernall, Frank Park, Cecilia J. Hillard, Pal Pacher

**Affiliations:** ^1^ School of Pharmacy, The Institute for Drug Research, The Hebrew University of Jerusalem, Jerusalem, Israel; ^2^ Roche Innovation Center Basel, F. Hoffman-La Roche Ltd., Basel, Switzerland; ^3^ Neuronal and Cellular Signal Transduction, Center for Molecular Neurobiology Hamburg (ZMNH), University Medical Center Hamburg-Eppendorf, Hamburg, Germany; ^4^ Department of Chemistry, University of Otago, Dunedin, New Zealand; ^5^ Department of Pharmaceutical Sciences, College of Pharmacy, University of Tennessee Health Science Center, Memphis, TN, United States; ^6^ Department of Pharmacology and Toxicology, Neuroscience Research Center, Medical College of Wisconsin, Milwaukee, WI, United States; ^7^ Laboratory of Cardiovascular Physiology and Tissue Injury, National Institute of Health/NIAAA, Rockville, MD, United States

**Keywords:** CB2R, CB2R ligands, inflammation, neurodegenarative diseases, addiction

The cannabinoid receptor type 2 (CB2R) has emerged as a promising therapeutic target for treating various pathologies. Under normal conditions, CB2R is primarily expressed in the immune system, but there is emerging evidence that various states of disease can lead to robust induction of this receptor. This suggests that CB2R is a viable therapeutic target and for this reason, molecules interacting with CB2R have been tested as potential treatments in a wide array of chronic conditions, including cardiovascular and gastrointestinal/inflammatory bowel disease; liver, kidney, lung, neuro-degenerative and psychiatric disorders; reproductive system and skin pathologies; inflammation; pain; cancer; and osteoporosis (Whiting et al., 2022). Through the years, researchers have designed and synthesized novel ligands targeting CB2R with a preference to be highly selective over the cannabinoid receptor type 1 (CB1R) to avoid undesirable CB1-dependent psychotropic effects. However, the clinical results using these CB2R ligands have been largely ineffective (Morales et al., 2016; An et al., 2020).

Greater knowledge of ligand-target binding kinetics, CB2R biased signaling and allosterism, and additional structures of antagonist- and agonist bound CB2R will likely enable more selective drug design (Soethoudt et al., 2017). This will bring new hope for the therapeutic potential of CB2R and a better understanding of the endocannabinoid system (ECS).

This Research Topic provides more insight into our current understanding of the CB2R field and its therapeutic potential and highlights new findings.

Four comprehensive reviews cover diverse aspects of the therapeutic potential of CB2R. Hashiesh et al. provide a full overview of the pharmacological properties, molecular and signaling mechanisms, and therapeutic potential of the CB2R specific agonist JWH133 in various pathological conditions. This review provides confirmation that CB2R is a viable therapeutic target, but that more preclinical pharmacokinetic and safety data is needed to develop effective human treatments. Young and Denovan-Wright thoroughly review the role of microglia and the ECS in neuroinflammation. Observed variations regarding components of the ECS in microglia together with the potential of CB2R as a therapeutic target are discussed. In the review by Liu et al., the authors propose that specific agonists of CB2R may serve as disease modifiers in type 1 diabetes. They demonstrate the involvement of CB2R in regulating the inflammasome and controlling intracellular autophagy, governing the secretion of extracellular vesicles from adipocytes and thus, dysregulating which induces chronic inflammation and obesity. In this regard, CB2R activation may play a similar role in the islets of Langerhans. Naturally occurring CB2R selective agonists or selective, peripherally restricted synthetic cannabinoids that work by intervening in both CB1R and CB2R signaling needs further investigation. The review by Franco et al. discusses the binding mode at orthosteric sites and/or exosites underlying the therapeutic potential of drugs targeting CB2R. According to the authors, a drug in a specific CB2R conformation leads to a signaling cascade that differs qualitatively and/or quantitatively from that triggered by another drug. A given drug may lead to different signaling outputs in a cell- or tissue-dependent manner due to potentially distinct allosteric effects from unique interactions with other proteins or with membrane lipids on the receptor. This highlights the pharmacological complexity of this receptor and the need to further unravel the binding mode of CB2R ligands in order to fine-tune signaling effects and therapeutic propositions.

A research article by Simard et al. provided data on the expression of both CB1R and CB2R in human blood leukocytes. The expression of CB2 mRNA can be detected in eosinophils, neutrophils, monocytes, and B and T lymphocytes, with the highest abundance in human eosinophils and B lymphocytes. The authors also review the evidence obtained from primary human leukocytes and immortalized cell lines regarding the regulation of their functions by CB2R, which highlights the urgent need to deepen the understanding of CB2R as an immunoregulator in humans.

Previous research proved that CB2R expression in the CNS is low under physiological conditions and is elevated in chronic neuroinflammatory states associated with neurodegenerative diseases. Esteban et al. analyzed the expression of CB2R in cortical areas of the brain of an AD mouse model (5xFAD/CB_2_^EGFP/f/f^) and showed that CB2Rs are expressed in the dystrophic neurite-associated microglia and their modulation modifies the number and activity of microglial cells as well as the metabolism of the insoluble form of the amyloid peptide. Thus, microglial CB2Rs can be potential targets for the development of amyloid-modulating therapies.

Brain CB2Rs were shown to be involved in drug reward and addiction. Indeed, He et al. reported that β-caryophyllene (BCP), a natural CB2R agonist, has therapeutic effects on methamphetamine (METH) abuse and dependence. Systematic administration of BCP dose-dependently inhibited METH self-administration in rats, indicating that BCP reduces METH reward, METH intake, and incentive motivation to seek and take METH.

A study by Reichenbach et al. demonstrated that CB2R ligands can influence the antinociceptive effects of morphine. The authors provide evidence of interactions between the CB2R selective agonist O-1966 and morphine that are probably mediated in part by the direct binding activity of O-1966 on the mu-opioid receptor. This interaction results in decreased potency of morphine to produce acute thermal antinociceptive effects, but can also lead to the potentiation of morphine antinociceptive tolerance, suggesting complex alterations in morphine signaling. However, O-1966 co-administration also blocked morphine hyperalgesia, and led to an attenuation of morphine tolerance when administration followed each morphine injection, perhaps due to well-documented and anti-inflammatory effects of CB2R agonism.

Keller et al. focused their study on p62 (sequestosome 1, SQSTM1) as an interaction partner for CB2R. In their research, JWH133 resulted in a weak osteoanabolic function in mice. Furthermore, this CB2R agonist modulated the bone cell differentiation in p62 KO animals comparable to Paget´s disease of bone indicating that p62 influences the function of CB2R. The authors emphasize the need for more studies to explore the possibility that this molecular link affects bone processes under pathological conditions or at older ages and is thus involved in disorganized bone turnover or osteoclast activity.

Ribeiro et al. demonstrated in their research article that the antidepressant-like behavior and the pro-neurogenic effect promoted by escitalopram (Esc) in stressed mice are in part mediated by CB2Rs. The chronic reduction of endogenous CB2R activity by the CB2 inverse agonist, AM630, attenuated the neuroplastic, the antidepressant- but not the anxiolytic-like effects of Esc.

Jayarajan et al. found that O-1966 inhibits allogeneic skin graft rejection *in vivo* supporting the fact that CB2R selective agonists may have the potential to act as a new class of compounds to prolong graft survival in transplant patients.

A theoretical study by El-Atawneh and Goldblum was used to build activity models for CB2R and other targets such as CB1R, peroxisome proliferator-activated receptor gamma (PPARγ), and 5-hydroxytryptamine receptor 4 (5-HT4R) for combinations that could be used for various indications such as Inflammatory Bowel Disease (IBD). Many dual CB2R/CB1R agonists were found together with CB2R agonists that acted also as 5-HT4R agonists. The authors also performed CB2R docking studies and found lower statistical performance of the docking (“structure-based”) compared to “Iterative Stochastic Elimination” ISE modeling (“ligand-based”) suggesting that ISE modeling may be a better starting point for molecular discovery than docking.

Despite significant progress in CB2R research, including the studies reported in this Research Topic, several hurdles toward a CB2R-based therapy remain to be cleared. Detection of CB2R protein still represents a major challenge for researchers. There is an essential need for simultaneous use of multiple approaches to confirm the expression of CB2R in cells/tissues (e.g. RNA sequencing, digital droplet PCR, RT-PCR, RNA-scope, new fluorescent probes, radioligand binding, PET-CT with radioligands, etc) including proper positive and negative controls. The use of CB2R antibodies is not recommended in tissues. Thus, it is very difficult to do proper target validation of CB2R in diseases and consequently in clinical trials. Regarding CB2 agonists, most of the CB2-related therapeutic conclusions are based on the effects of nonselective and nonspecific first-generation ligands (JWH133, AM1241, and AM630, etc) and have not been confirmed with more selective ligands. Numerous problems exist with the first generation of commercially available ligands: 1) Selectivity and specificity issues (numerous off-targets and potential effect on CB1R *in vivo*), 2) Few of the ligands used were tested on mouse CB2Rs where the binding is often decreased (compared to humans); in some cases, the ligands, may even exert opposite effects on human vs. mouse receptors (e.g. agonist vs. inverse agonists), 3) These ligands have less than optimal bioavailability (e.g. short half-life, rapid degradation in the liver, etc), which is often ignored in the study designs, making the conclusions questionable, 4) The quality control is not good (degradation and contamination with endotoxins, and organic solvents are possible), and 5) Some of the ligands have biased signaling on CB2R, hence, introducing another layer of complexity in understanding the therapeutic effects/potential of these ligands. Furthermore, many studies conclude a role for CB2R in behavioral or other CNS-mediated effects based upon antagonism by SR144528. However, this compound has very poor brain penetrance, which complicates the interpretation of these studies.

Thus, better tools and multiple approaches using proper positive and negative controls are required to evaluate the CB2R expression in normal and pathological tissues in order to succeed with the target validation in preclinical and clinical studies/trials. Development of more selective and specific ligands with better PK properties and known effects on CB2R signaling (in mice, rats, primates, or humans) are required. The use of multiple validated approaches for CB2R detection, in concert with the new generation of CB2R ligands and genetic tools (e.g. tissue and cell specific CB2R knockouts, and GFP mice, etc) could enhance our understanding of the role of CB2R signaling in health and disease and facilitate development of successful therapies to ease human suffering.
